# How to Surge to Face the SARS-CoV-2 Outbreak: Lessons Learned From Lombardy, Italy

**DOI:** 10.1017/dmp.2020.64

**Published:** 2020-04-01

**Authors:** Roberto Faccincani, Federico Pascucci, Sten Lennquist

**Affiliations:** Ospedale San Raffaele, Emergency Department, Milano, Italy; Ospedale San Raffaele, UniSR, Milano, Italy; Linköping University, Professor Emeritus, Linkoping, Sweden

**Keywords:** disease outbreaks, novel coronavirus, pandemia, severe acute respiratory syndrome, surge capacity

## Abstract

Italy is fighting against one of the worst medical emergency since the 1918 Spanish Flu. Pressure on the hospitals is tremendous. As for official data on March 14th: 8372 admitted in hospitals, 1518 in intensive care units, 1441 deaths (175 more than the day before). Unfortunately, hospitals are not prepared: even where a plan for massive influx of patients is present, it usually focuses on sudden onset disaster trauma victims (the most probable case scenario), and it has not been tested, validated, or propagated to the staff. Despite this, the All Hazards Approach for management of major incidents and disasters is still valid and the “4S” theory (staff, stuff, structure, systems) for surge capacity can be guidance to respond to this disaster.

Since the first case of novel coronavirus (2019-nCoV) was recognized in Codogno, Lombardy, on 20 February 2020,^1^ Italy has been fighting against one of the worst medical emergency since the H1N1 influenza pandemic in 1918, known as the Spanish Flu, which was responsible for around 500,000 deaths.^2^

In an attempt to contain the contagion and, therefore, the surge in health-care demand, the Italian Government has imposed the most restrictive measures to people’s freedom of movement and socialization since the martial state during WWII: schools are closed, sport and recreating activities in groups are forbidden, movements are restricted to documented essential needs.

The significance of the 17,750 positive 2019-nCoV tests (2795 more than the day before) declared by the Italian Government on 14 March 2020 is unclear: tests are different (nasopharyngeal and oropharyngeal swab; bronchoalveolar lavage; endotracheal aspirate, nasopharyngeal or nasal wash/aspirate…), with different sensitivity.^3^ In addition, there are times when the results come 2-3 days after the sample was taken and people were unaware of their positive status and transmitted the virus. Most importantly, the number of people positive while asymptomatic or with minor symptoms is not negligible.

Pressure on the hospitals is tremendous; as for official data on 14 March 2020, 8372 admitted in hospitals, 1518 in intensive care units (ICUs), 1441 deaths (175 more than the day before). Also, we know very little about people who do not present to hospitals. This is without a doubt a medical major emergency/disaster and should be managed accordingly.

Unfortunately, hospitals are not prepared. Even where a plan for massive influx of patients is present, it usually focuses on sudden onset disaster trauma victims (the most probable case scenario), and it has not been tested, validated or propagated to the staff.^4^ Despite this, the All Hazards Approach (AHA) for management of major incidents and disasters is still valid.^5^

## THE 4S THEORY OF SURGE CAPACITY

This is the 4S theory^6^ of surge capacity applied to this pandemic.

### Staff

Specialized staff will be one of the biggest bottlenecks: emergency department (ED), ICU, and infectious disease personnel will be most needed. Other medical staff will likely have their practices curtailed or postponed and their practice locations will be made available for 2019-nCoV patients. These physicians can then be repurposed to attend to 2019-nCoV patients or others outside their normal practice, with early training methods mixing specialized and nonspecialized staff during the early phases of the response.

Heavy PPE (personal protective equipment (PPE), to be changed for every patient, will likely make the staff exhausted. It is advisable to plan for short shifts with adequate rest.

### Stuff

Adequate PPE is mandatory to protect the staff and to avoid the spread of the 2019-nCoV inside the hospital. The consumption of PPE will be massive, so it is recommended to make big stockpiles and to plan for emergency provision. A clear policy on PPE will also be needed, because it is predictable that not only the public but also the health professionals will tend to overestimate their risk of infection and over-use.^7^

Oximeters, O2 masks, O2 delivery points, NIV (non-invasive ventilation) devices and ventilators will be soon saturated. It is recommended to anticipate the requests. A good idea could be having an inventory of the available devices. Ventilators will become a bottleneck very early. Find the next ventilator before having the next patient in need.

### Structure

Reconfiguration of the hospital according to the activities and intensity of care is advisable. The 2019-nCoV diagnostic and treatment activities will require negative pressure rooms (including operating theaters and delivery rooms). Chest X-ray and lung computed tomography (CT) scan facilities should be increased in number as soon as possible. It is also essential to develop new temporary (in the ED) or definitive sites of care with different intensity: normal wards with just O2 delivery points, sub-intensive departments for NIV, and ICUs.

Identify in advance alternative sites of care. Outpatient clinics could be easily converted to expand the capacity of the ED (availability of oxygen delivery points is the only real limitation); operating theaters are excellent supplementary ICUs. It is advisable to not spread the same activity in many different places, far from one another, but to keep all the 2019-nCoV patients centralized. In practice, 10 ICU beds can easily be managed by 1 intensivist with 4-5 ICU nurses if in one single open space, while the same 10 ICU beds will need double staff if split in 2 different rooms.

If at all possible particular attention should be paid in separating patients 2019-nCoV positive and negative. Creating separate pathways before and after the identification of the 2019-nCoV positivity could be the solution.

Pregnant women, children, and people with disability should also have as well their own pathways.

Patients waiting for the result of the 2019-nCoV test are an important “new category.” If these patients are in a life-threatening condition, they will be managed as positive for 2019-nCoV. In the other cases, they are in a sort of limbo, and mixing them with the already known positive and negative should be absolutely avoided. This means that they will get stuck in the ED, or you will have to isolate them if you discover a risk in the ward. The 2019-nCoV testing activity could easily become a bottleneck. To avoid this possibility, this activity should work 24/7, at the maximum capacity. In addition, a clear policy on 2019-nCoV testing is mandatory to avoid incorrect indications.

The staff should consider all the patients as potentially 2019-nCoV positive until proven otherwise, although clinically at low risk. All the staff of the ED should wear the highest level available of PPE against droplets and contact transmission and change it for each patient. PPE against air transmission should be worn in case of invasive procedures, in particular the ones necessary for airway management.

The *triage area* in the ED should aim to select people with high or low probability to have 2019-n-CoV infection, as well as to sort out priority to treatment. It could be effective to move the triage area out of the entrance of the ED and to arrange separate pathways for the different risk categories. It will be almost impossible to have 100% of specificity in recognizing truly positive and negative cases on a clinical basis, and this will divide your experienced triage staff. Clear triage protocols and effective training to learn how to use them will increase sensitivity.

Creating different investigation/treatment areas for patients at high or low risk is a good solution to decrease the spread of the infection. Among the high-risk ones (the majority), identify separate locations for people with the different priorities: O2, NIV, intubation. Always be ready to move patients from an area to another one, considering that some patients deteriorate very quickly and other ones show the typical 2019-nCoV symptoms while being in the non–2019-nCoV areas.

Unfortunately, it is better to be prepared for an increase of fatalities and for fatality management. Visits by relatives were forbidden for the risk of contagion, it might be useful to have a room in the morgue with the possibility to see the deceased through windows. Regarding the need of autopsy of a 2019-nCoV positive cadaver, it is recommended to do it in a negative pressure room and to avoid aerosol generating procedures. The use of full PPE is mandatory.

### Systems

A clear command chain is compulsory. It starts with people in command 24/7 for each area of the different activities. It appeared effective to have an overall 24/7 commander of the operational activities. This essential figure should manage patient flow, know the needs of all the different areas, and re-allocate resources. This commander of the operations has to stay in constant contact with the Hospital Command Group, a more strategic level where the medical expertise should be flanked by logistic, administrative, maintenance, and communication experts.

An effective communication strategy should be implemented both inside and outside the hospital. The ability to pass information among the hospital staff, to transmit down/up needs and requests, and to communicate availability to accept new patients or difficulties in capacity is of the utmost importance. It is also essential to receive information by the emergency medical services regarding their needs, and to offer the capacity or to communicate oversaturation of the ED and hospital.

Communication strategy with regard to media is also very sensitive and must be considered carefully.

Communication with relatives and caregivers is also a concern in a pandemic. Relatives and caregivers should be asked to go home and stay in quarantine until further information. Pay attention to have a secure contact and to inform them as soon as news about the patient are available. Ensure them that the needed public health measures will be taken according to the result of the investigations. When patients are able, invite them to keep timely contacts with their relatives through mobile phones.

People already in isolation, as well as health professionals under pressure and scared to get the infection and to take it home, can easily develop psychological disorders. Psychological support should be made available and the service richly advertised.

Anticipate and not overrun the needs. There will be a huge consumption of PPE and you cannot risk running out. Staff will eventually get sick, and you must be ready to replace a not negligible number of personnel (mix the specialized and nonspecialized staff very early). A systematic organization to monitor respiratory deterioration should be established at every level.

Produce protocols for triage, diagnosis, and treatment and train the staff.

Plan for ethical consideration regarding treatment plans with patients to match the best care for that patient and the best care for other patients.
